# Electrochemical Deposition of Gold Nanoparticles on Reduced Graphene Oxide by Fast Scan Cyclic Voltammetry for the Sensitive Determination of As(III)

**DOI:** 10.3390/nano9010041

**Published:** 2018-12-29

**Authors:** Guo Zhao, Gang Liu

**Affiliations:** 1 Key Laboratory of Modern Precision Agriculture System Integration Research, Ministry of Education of China, China Agricultural University, Beijing 100083, China; guozhao1989@gmail.com; 2 Key Laboratory of Agricultural Information Acquisition Technology, Ministry of Agriculture of China, China Agricultural University, Beijing 100083, China

**Keywords:** reduced graphene oxide, gold nanoparticle, square wave anodic stripping voltammetry, arsenic detection, soil

## Abstract

In this study, a stable, sensitive electrochemical sensor was fabricated by the electrochemical codeposition of reduced graphene oxide (rGO) and gold nanoparticles on a glassy carbon electrode (rGO-Au_nano_/GCE) using cyclic voltammetry (CV), which enabled a simple and controllable electrode modification strategy for the determination of trace As(III) by square wave anodic stripping voltammetry (SWASV). SWASV, CV, electrochemical impedance spectroscopy (EIS), X-ray diffraction (XRD) and scanning electron microscopy (SEM) were used to characterize the electrochemical properties and morphology of the proposed sensing platform. The number of sweep segments, the deposition potential and the deposition time were optimized to obtain ideal sensitivity. The presence of rGO from the electroreduction of graphene oxide on the sensing interface effectively enlarged the specific surface area and consequently improved the preconcentration capacity for As(III). The rGO-Au_nano_/GCE sensor exhibited outstanding detection performance for As(III) due to the combined effect of Au_nano_ and rGO formed during the electroreduction process. Under the optimized conditions, a linear range from 13.375 × 10^−9^ to 668.75 × 10^−9^ mol/L (1.0 to 50.0 μg/L) was obtained with a detection limit of 1.07 × 10^−9^ mol/L (0.08 μg/L) (S/N = 3). The reproducibility and reliability of the rGO-Au_nano_/GCE sensor were also verified by performing 8 repetitive measurements. Finally, the rGO-Au_nano_/GCE sensor was used for the analysis of real samples with satisfactory results.

## 1. Introduction

While As(III) is one of the most toxic forms of arsenic, even at low concentrations, it is widespread in natural environments [1,2]. As(III) contamination in agricultural soil is a serious problem because the presence of As in the food chain can cause many health problems [3,4], such as bladder cancer, lung cancer, keratosis and skin lesions. Therefore, the development of a simple, fast and sensitive method for the determination of As(III) in soil is urgently needed. Generally, there are two types of analytical strategies for the detection of As(III): spectroscopic and electrochemical methods. Spectroscopic methods, such as inductively coupled plasma mass spectrometry [5], atomic absorption spectroscopy [6] and hydride generation atomic fluorescence spectrometry [7], have very high detection accuracy. However, these methods require expensive instruments, long analysis times, and laboratory conditions. They are not suitable for on-site analyses and routine monitoring, especially for large numbers of samples. In contrast, one electrochemical method, anodic stripping voltammetry (ASV), has been widely used for trace analyses of metal ions in different environments and industry samples due to its outstanding analytical performance, low cost, convenient operation and high sensitivity. ASV mainly consists of two steps [8]: preconcentration and stripping. The target metals are electrodeposited on the working electrode surface under a reduction potential in the preconcentration step. Then, the metals preconcentrated on the electrode surface are oxidized into their cationic forms under a scanning potential, and the concentration of the target heavy metal is proportional to the stripping response current. Therefore, the working electrode substrate plays a key role in improving the detection sensitivity of this technique.

Dropping mercury electrodes have frequently been used with stripping voltammetry for the analysis of heavy metals due to their high sensitivity and reproducibility. However, because of their operational limitations and potential toxicity, electrochemical sensors that use dropping mercury electrodes as the working electrode have been gradually replaced by sensors with solid electrodes. ASV with gold nanomaterial-modified electrodes has been widely applied for the determination of As(III) [9,10], and gold has already been verified to improve the sensitivity of a bare electrode by the electrogeneration of H_2_ [11]. Gold nanoparticles can be made by chemical synthesis [12,13], ultraviolet (UV) light [14], electron-beam irradiation [15] or electrochemical methods [16,17].

Some reports have indicated the cathodic formation of a single As(0) monolayer on an Au surface, possibly due to the nonconducting nature of As(0) deposits. Because As(0) deposits are nonconductive, increasing the specific surface area is very important to improve the ASV analysis sensitivity for As(III). Li et al. reported a facile and green approach for fabricating Au-reduced graphene oxide (Au-rGO) nanocomposites by UV irradiation to detect As(III) [14]. The mixture of GO and HAuCl_4_ was irradiated with a high-intensity UV spot lamp, while nitrogen was bubbled through the solution to activate the reduction reaction. After 20 min of irradiation, the product was separated by centrifugation and washed with water. Next, the sample was redispersed in water and used to modify an electrode surface for the detection of As(III). Electrodeposition is a cost-effective approach to rapidly and directly grow Au nanoparticles in an aqueous solution. The interplay between the crystal growth rate and the mass transport rate in the electrocrystallization can be readily manipulated via the control of deposition potential without changing the reactant concentration [18,19,20]. Recently, an electrochemical reduction strategy was developed and further applied to the modification of graphene using graphene oxide (GO). The results demonstrated that GO can be reduced on the surface of an electrode to form one or several graphene layers with a controllable potential, such as those used in cyclic voltammetry (CV) and potentiostatic methods [21]. Hence, based on the discussion above, the preparation and modification of reduced GO (rGO) and Au_nano_ by a one-step coelectrodeposition process to form a rGO-Au_nano_ sensing interface is possible because under cathodic conditions, both metal ions and GO have the ability to obtain electrons and activate reduction reactions, as reported in reference [22]. Additionally, the thickness of the Au-rGO nanocomposite film formed on the electrode surface can be controlled by the deposition time. After the optimization of the deposition time, the total time needed for the preparation of nanocomposite and modification of the electrode is 120 s, which is far less than the time for the ultraviolet irradiation method. Clearly, compared to the ultraviolet irradiation method electrochemical deposition is a fast, easy, convenient and effective method that does not require special instruments for the preparation of Au-rGO nanocomposite films [23].

In this study, we attempted to develop a rGO-Au_nano_ nanocomposite with a high specific surface area and excellent catalytic performance using solution-dispersed GO and HAuCl_4_ with CV, which is a controllable technique that produces sensitive, homogeneous and stable sensing interfaces. The proposed rGO-Au_nano_-modified glassy carbon electrode (GCE) exhibited high sensitivity and stability due to the combined effect of the rGO and Au_nano_ formed via electrochemical reduction, outstanding catalytic ability for As(III) and good electrical conductivity. The electrochemical analysis parameters, i.e., the number of sweep segments, deposition potential and deposition time, were optimized. Interference from nontarget heavy metals and the repeatability of the developed electrochemical platform were also investigated. Finally, the fabricated rGO-Au_nano_/GCE was applied for the analysis of trace As(III) in real soil samples by SWASV with satisfactory results.

## 2. Materials and Methods

### 2.1. Materials

HAuCl_4_ was purchased from Sinopharm Chemical Reagent Co., Ltd. (c). Arsenic trioxide (As_2_O_3_) was purchased from Aldrich (Sigma-Aldrich, Saint Louis, MO, USA) and then dissolved in 1.0 M aqueous NaOH to create a 1000 mg/L solution. GO was obtained from Nanjing JCNANO Materials Tech Co., Ltd. (Nanjing, China). A nitric acid solution (1.0 M) was used as the supporting electrolyte during the stripping voltammetry analysis of As(III). Millipore-Q (18.2 MΩ) water was used for all experiments.

### 2.2. Instrument

Scanning electron microscopy (SEM) was performed on a NovaNanoSEM 450 scanning electron microscope. X-ray diffraction (XRD) patterns of Au_nano_ were obtained with a PANalytical Empyrean Series 2 X-ray diffractometer in the 2θ range from 30° to 90°. The electrochemical measurements and analyses, such as electrochemical impedance spectroscopy (EIS), CV and SWASV, were carried out using a CHI660D electrochemical workstation (Shanghai CH Instruments, Shanghai, China). A Pt wire electrode, Ag/AgCl electrode and GCE (ø3 mm) were used as the counter, reference and working electrodes, respectively, to construct a three-electrode system. A 25 mL beaker was used as the electrolytic cell for all electrochemical measurements. A magnetic stirrer was used to stir the test solution during the deposition step.

### 2.3. Synthesis and Modification of the rGO-Au_nano_ Nanocomposite

Prior to modification, the GCE surface was polished using 0.05 mm alumina powder, and the electrode was then sonicated in 1:1 HNO_3_, absolute ethanol and water in sequence to remove alumina residue. GO was dispersed in a PBS solution (0.1 M, pH 9.0) by ultrasonication to form a GO dispersion with a concentration of 0.1 mg/mL. HAuCl_4_ was added to the GO suspension to form 0.1 mM Au^3+^, and then nitrogen purging was performed for 1 h to deoxygenate the solution and exfoliate the stacked GO. CV electrodeposition was carried out at a scan rate of 50 mV/s in the potential range from −1.4 to 0.6 V for 10 sweep segments (i.e., 5 cycles). After electrodeposition, the rGO-Au_nano_/GCE was gently washed with ultrapure water. The same procedures described above were used to prepare the other electrodes.

### 2.4. Electrochemical Detection of As(III)

The SWASV measurements for As(III) detection were carried out based on a three-electrode system that used the rGO-Au_nano_/GCE as the working electrode under the optimized conditions. A deposition potential of −0.3 V vs. Ag/AgCl was applied to the as-prepared rGO-Au_nano_/GCE for 250 s in a nitric acid solution (0.1 M) with stirring during the deposition step. After equilibration for 10 s, the stripping voltammogram was recorded from −0.2 to 0.4 V vs. Ag/AgCl with a step potential of 5 mV, a pulse amplitude of 25 mV and a frequency of 25 Hz. An activation process was initiated in the nitric acid solution (0.1 M) to clean the electrode surface and remove previous residual As(0) using a constant positive potential of 0.6 V for 120 s. For comparison, the same process and conditions were used for the other modified electrodes.

### 2.5. Soil Sample Preparation

The soil samples were obtained from farmland in China. The detailed process is as follows: first, 40 mL of 0.1 M nitric acid was mixed with a 10 g soil sample using strong shaking. Then, a portable ultrasonic extractor was used to sonicate the above mixture for 10 min. After that, the obtained mixed solution was centrifuged (2000 rpm) using a portable centrifuge, and then, 20 mL of supernatant was loaded into a 30 mL electrolyte cell for the SWASV measurements. Krasnodêbska-Ostrêga and Kowalska [24] verified that there is no significant difference between the ultrasonic-assisted extraction method and the method proposed by the European Communities Bureau of Reference.

## 3. Results and Discussion

### 3.1. Electrochemical Deposition of the rGO-Au_nano_ Composite

**Figure 1:**
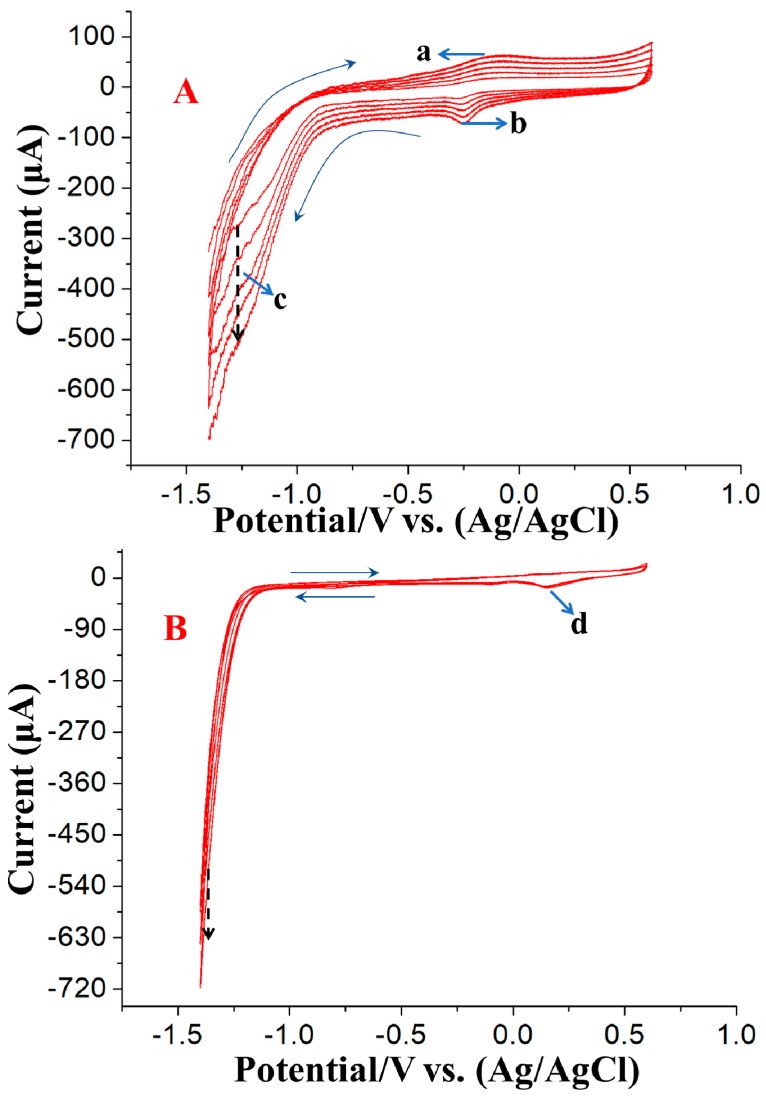

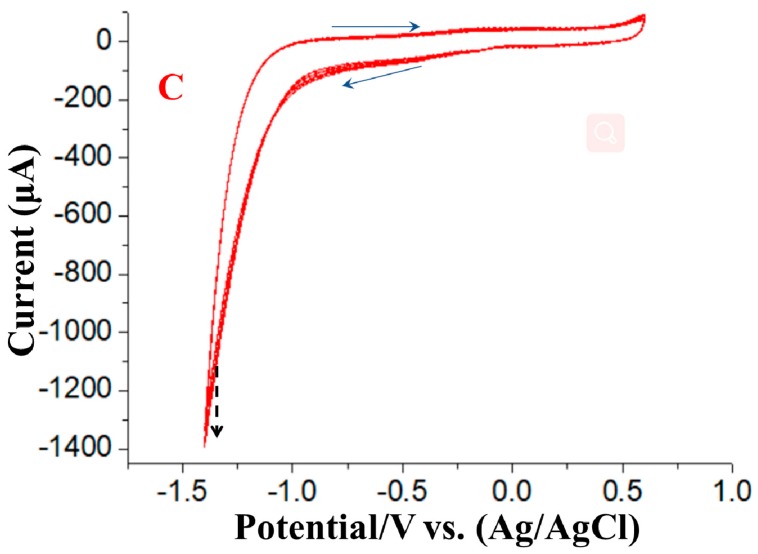
CV curves for the electroanalysis of (**A**) 0.1 mg/mL GO, (**B**) 0.1 mM HAuCl_4_ and (**C**) 0.1 mg/mL GO + 0.1 mM HAuCl_4_ in pH 9.0 PBS buffer solution at a scan rate of 50 mV/s. The black dash arrow and blue arrow indicate the scan direction and current changes direction, respectively.

The CV electroanalysis curves of GO on a GCE can be seen in Figure 1A, and one oxidation peak (a) and two reduction peaks (b and c) can be observed. The peak currents increased with successive potential scans, demonstrating that GO was successfully electrochemically reduced on the electrode surface. The current peak (c) was due to the reduction of the oxygen-containing groups on GO [25,26,27]. The peak currents that appeared at positions “a” and “b” were due to the redox reaction of some redox pairs on the graphene plane, and peak “c” was attributed to the irreversible electrochemical reduction of GO [28].

The CV curve for the electroanalysis of 0.1 mM HAuCl_4_ is shown in Figure 1B. A significant reduction peak is present at approximately 0.18 V, indicating that the gold salt was reduced from Au^3+^ to Au^0^. The electrochemical reduction of Au_nano_ on the electrode surface would be an outstanding active site for the reduction of Au^3+^ to Au^0^. The CV electroanalysis results for the mixture of GO and HAuCl_4_ are shown in Figure 1C, and these results were completely different from those observed for the GO electroanalysis. One significant difference is that the currents from scanning at a positive potential to scanning at a negative potential in the range of −1.2 V to −1.4 V for the mixture of GO and HAuCl_4_ were dramatically larger than those obtained the GO electroanalysis, indicating the continuous electrodeposition of Au_nano_, which is more conductive than rGO, onto the electrode surface.

### 3.2. Characteristics of the Modified Electrodes

**Figure 2:**
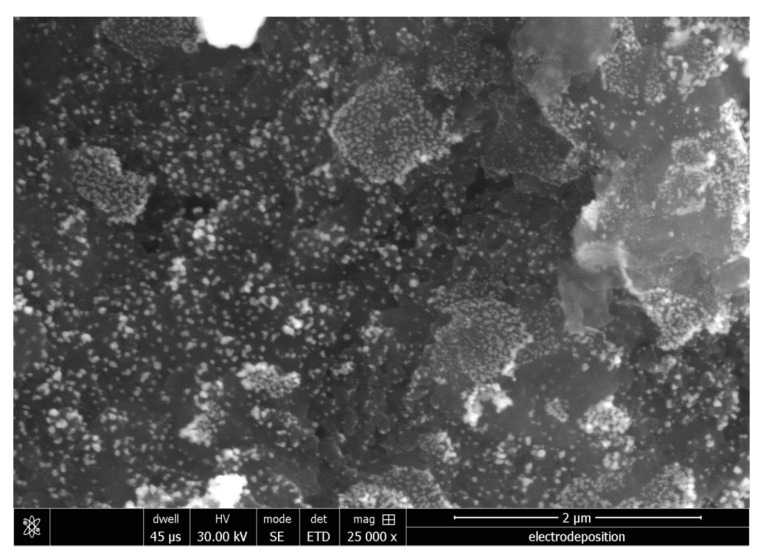
SEM image of rGO-Au_nano_/GCE.

As shown in Figure 2, SEM was used to characterize the morphology of the rGO-Au_nano_ sensing interface formed by electrochemical reduction. SEM imaging of the rGO-Au_nano_ deposited on a GCE showed a large amount of Au_nano_ evenly distributed on the substrate, which had a wrinkled texture associated with the presence of rGO sheets. The uniform distribution of Au_nano_ with an average diameter of ~30 nm indicated that rGO was formed with Au_nano_ by the electrochemical deposition, which effectively promoted the specific surface area of the sensing interface and allowed a large amount of Au_nano_ to be deposited on the electrode surface.

**Figure 3:**
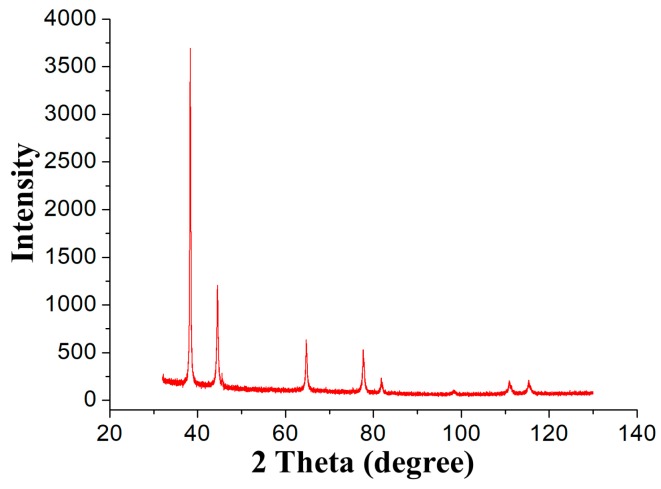
Representative XRD pattern of Au_nano_ synthesized by electrodeposition in a 0.1 mM HAuCl_4_ solution.

As shown in Figure 3, XRD analysis was carried out, and the XRD pattern was analyzed to investigate the monocrystalline nature of the nanoparticles formed by the electrochemical reduction of the gold salt. Four diffraction peaks appeared at 2θ = 38.2°, 44.5°, 65.6° and 78.6° in the XRD pattern, which were consistent with the (111), (200), (220) and (311) planes, respectively, of gold metal (International Center for Diffraction Data, ICDD No. 4-0783), suggesting that the product synthesized by electrodeposition contains pure crystalline gold. The Bragg reflection peaks of Au_nano_ clearly indicate a face-centered cubic monocrystalline structure, which is consistent with the crystalline structure of gold. The XRD pattern shows a very high Bragg reflection peak corresponding to the (111) lattice plane, indicating that Au_nano_ related to the (111) lattice plane was flat [29] on the planar surface.

**Figure 4:**
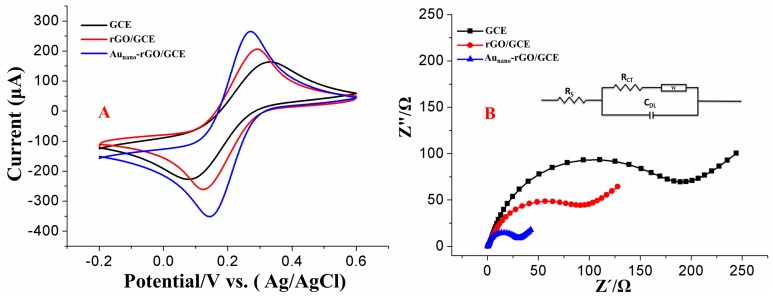
CV (**A**) and EIS (**B**) results for bare GCE, rGO/GCE and rGO-Au_nano_/GCE in a solution of 5 mM [Fe(CN)_6_]^3−/4−^ containing 0.1 M KCl.

The electron transfer characteristics of the surfaces of different electrodes were studied by CV using [Fe(CN)_6_]^3−/4−^ redox probes, as shown in Figure 4A. The [Fe(CN)_6_]^3−/4−^ redox currents on rGO/GCE were larger than those on the bare GCE. Additionally, the potential difference between the oxidation peak and reduction peak of the [Fe(CN)_6_]^3−/4−^ redox probe decreased because of the good electron transfer capability of rGO. The redox currents of the redox probes were further enhanced on rGO-Au_nano_/GCE compared with those on bare GCE and rGO/GCE, which demonstrated that the presence of Au_nano_ in the rGO-Au_nano_ composite effectively improves the electron transfer capability.

In this study, EIS, a common electrochemical characterization method that consists of low- and high-frequency regions corresponding to semicircular and linear portions, respectively, was also used to further investigate the electron transfer kinetics of the different electrodes, as shown in Figure 4B. Additionally, an equivalent circuit was used to fit the EIS data, as shown in Figure 4B, where Rct, Rs and C_DL_ represent the charge transfer resistance, resistance of solution and double layer capacitance, respectively. Among the three parameters, Rct is the most important key factor and is widely used for the characterization of electron transfer kinetics on the electrode surface. The semicircular and linear portions represent the Rct and the surface diffusion process, respectively, which reflects the electron transfer kinetics of [Fe(CN)_6_]^3−/4−^ at the electrode interface [30]. A smaller, well-defined semicircle was observed in the high-frequency region with rGO/GCE than in the region of bare GCE, which suggested that the presence of rGO reduced the impedance of the electrode interface [31]. The smallest R_ct_ value was observed with rGO-Au_nano_/GCE because the corresponding diameter of the semicircle was the smallest, which suggested that the presence of Au_nano_ in the rGO layer can effectively improve the electron transfer kinetics of the electrode surface. The first EIS point of all the electrodes in the low-frequency region was almost the same, which indicated that Rs was the same for all the electrodes. These results were in good agreement with the CV results.

### 3.3. Optimization of the Experimental Conditions

**Figure 5:**
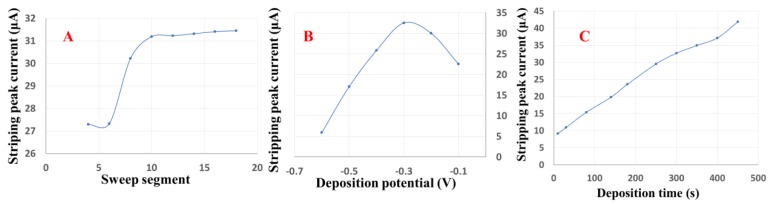
The effects of (**A**) the number of sweep segments, (**B**) the deposition potential and (**C**) the deposition time on the As(III) stripping peak currents on rGO-Au_nano_/GCE in a 0.1 M HNO_3_ solution containing 15 μg/L As(III).

In this study, the number of sweep segments, deposition time and deposition potential were investigated to determine the optimal experimental conditions for rGO-Au_nano_/GCE that result in the highest detection sensitivity of As(III). The effect of deposition time on the stripping response was investigated in a nitric acid solution (0.1 M). The thickness of the rGO-Au_nano_ sensing composite, which could have a significant effect on the sensitivity, can be controlled by changing the number of electrodeposition cycles, i.e., the number of sweep segments. In this work, 4 to 18 sweep segments were tested to optimize the thickness of the sensing film, and the results can be seen in Figure 5A, which shows the As(III) stripping response with respect to the number of electrodeposition sweep segments. The stripping response of As(III) significantly increased with the number of sweep segments in the range from 4 to 10. This increase may be because the GCE surface was not saturated with rGO-Au_nano_ until the number of sweep segments reached 10. As the number of sweep segments was increased above 10, the stripping response of As(III) remained nearly constant. Thus, 10 sweep segments were used for further experiments.

As shown in Figure 5B, the deposition potential was optimized for the As(III) stripping response in the potential range from −0.1 to −0.6 V with a deposition time of 250 s. At a deposition potential of −0.3 V, the highest peak currents were obtained; thus, a deposition potential of −0.3 V was used for further analyses.

Additionally, the effect of the deposition time on the stripping response was investigated in the range from 10 to 450 s, as shown in Figure 5C. The stripping signals of As(III) sharply increased as the deposition time increased. Finally, a deposition time of 250 s was chosen for the following measurements as a compromise between sensitivity and analysis time.

### 3.4. Stripping Responses of Different Electrodes

**Figure 6:**
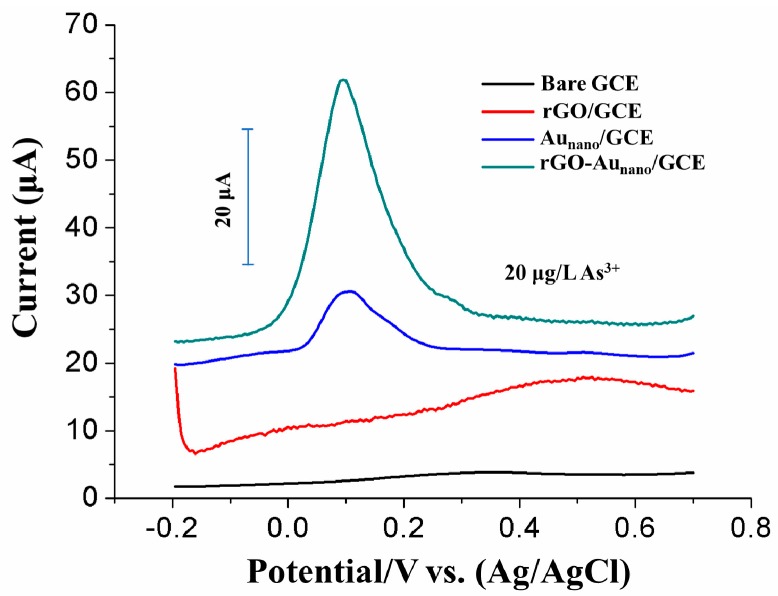
Comparison of SWASV curves for different modified electrodes used for the detection of 20 μg/L As(III) at a deposition potential of −0.3 V with a deposition time of 250 s in a 0.1 M nitric acid solution.

The stripping responses of different electrodes, i.e., a bare GCE, rGO/GCE, Au_nano_/GCE and rGO-Au_nano_/GCE, were investigated under the optimum conditions. As shown in Figure 6, As(III) produced no obvious response on bare GCE and rGO/GCE. However, a significant As(III) response can be observed on Au_nano_/GCE. A higher and sharper As(III) stripping response was obtained on rGO-Au_nano_/GCE. The enhanced stripping response of rGO-Au_nano_/GCE can be attributed to the following: (1) the electrodeposition of solution-state As(III) was enhanced due to the electrogeneration of H_2_ near Au_nano_ by chemical reduction [11]; (2) rGO increased the specific surface area and conductivity of the sensing interface; and (3) the combined effect of Au_nano_ and rGO synthesized by electrochemical reduction imparted a very high sensitivity for As(III) detection [14]. The results show that the rGO-Au_nano_ sensing interface-modified GCE has outstanding sensitivity and is highly promising for application.

### 3.5. Analytical Performance of rGO-Au_nano_/GCE

**Figure 7:**
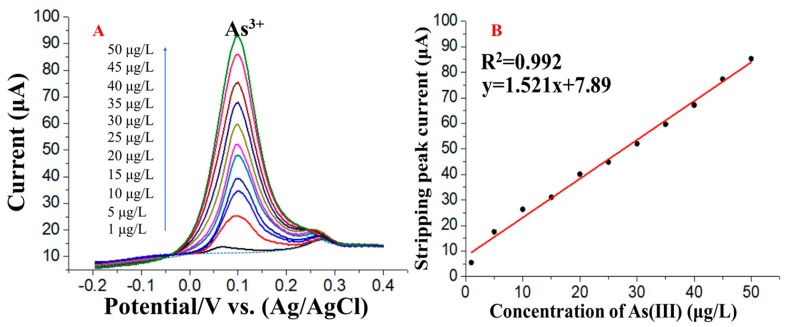
(**A**) SWASV response curves of rGO-Au_nano_/GCE for the detection of different concentrations of As(III) and (**B**) the corresponding relationship between concentration and peak current in the concentration range from 1 μg/L to 50 μg/L (deposition time: 250 s).

**Figure 8:**
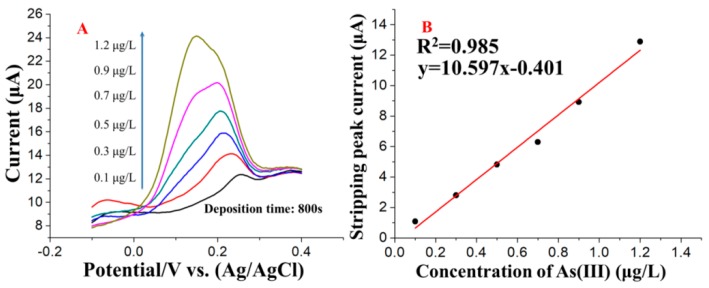
(**A**) SWASV response curves of rGO-Au_nano_/GCE for the detection of different concentrations of As(III) and (**B**) the corresponding relationship between concentration and peak current in a concentration range from 0.1 μg/L to 1.2 μg/L (deposition time: 800 s).

Under the optimal conditions, calibration curves for the stripping response of As(III) at both common and trace level concentrations were established. A series of SWASV As(III) responses and the corresponding calibration plots in the concentration range from 1 to 50 μg/L are shown in Figure 7A,B, respectively. The calibration curve and correlation coefficient were y = 1.521x + 7.89 (x: μg/L, y: μA) and 0.992, respectively. A significant and good linear relationship between the stripping peak current and the concentration of As(III) was obtained between 1.0 and 50.0 μg/L with a sensitivity of 1.521 μAμg/L. The SWASV As(III) responses in a lower concentration range, i.e., 0.1–1.2 μg/L, are shown in Figure 8A. The calibration curve and correlation coefficient were y = 10.597x − 0.401 (x: μg/L, y: μA) and 0.985, respectively. A detection limit of 0.08 μg/L was obtained according to the formula LOD = 3 σ/S, where σ is the standard deviation of a blank sample tested 10 times and S is the slope of the corresponding linear calibration equation of y = 1.521x + 7.89. The responses indicate that a higher sensitivity and lower LOD can be obtained by increasing the deposition time from 250 s to 800 s (Figure 8B). The specific stripping potential of As(III) changed as the As concentration changed from low to high. The peak shifting may be explained by the oxidation of additional As deposited on the electrode surface at a low positive potential with a high concentration of As(III). With a low concentration of As(III), only a trace amount of As was deposited on the electrode surface and was difficult to oxidize at a low positive potential; thus, the peak appeared at a more positive potential [32]. A comparison of the major analytical properties obtained in this work and those from previous work is shown in Table 1. Table 1 shows that the analytical performance of rGO-Au_nano_/GCE is comparable and even better than that in previous reports, and this electrode is more controllable and easier to prepare and use and offers a lower limit of detection than other previously reported electrodes.

### 3.6. Stability Measurements

**Figure 9:**
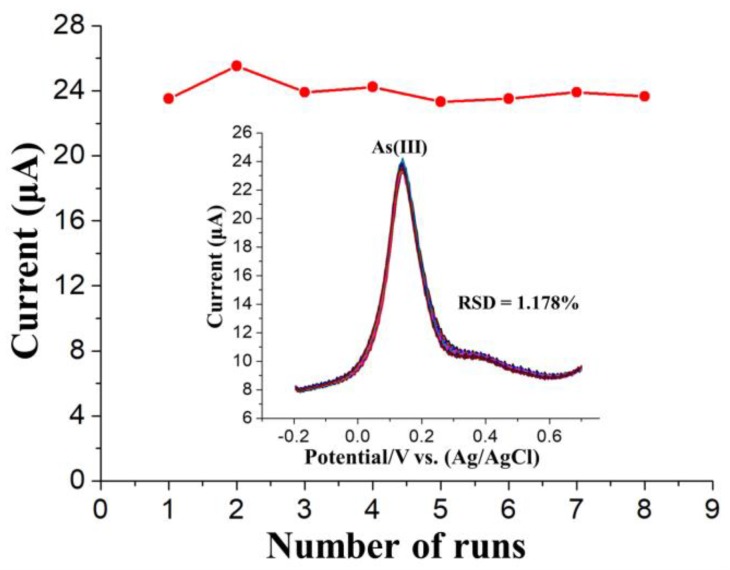
Eight repeated SWASV measurements of 5 μg/L As(III) with rGO-Au_nano_/GCE. The insets correspond to the data collected from every SWASV curve for a total of eight measurements. RSD refers to the relative standard deviation.

As shown in Figure 9, to verify the suitability and reproducibility of rGO-Au_nano_/GCE for As(III) determination, eight SWASV responses to 5 μg/L As(III) were obtained as continuous repetitive measurements. The times for each step were 250 s, 10 s, 4.8 s and 120 s for the deposition, standing, stripping and cleaning steps, respectively. Thus, the total working time of the electrode in the solution for each measurement should be 384.8 s. The relative standard deviation of the eight repetitive measurements of As(III) was 1.178%, which is less than 20%, demonstrating the good reusability and stability of rGO-Au_nano_/GCE for repeated SWASV measurements of As(III) under optimal conditions.

### 3.7. Interference Studies

**Figure 10:**
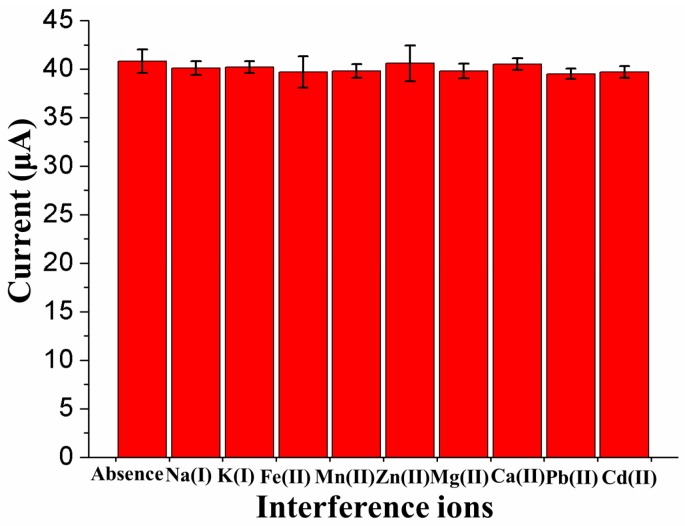
Interference study on the stripping peak currents of 15 μg/L As(III) in the presence of 10-fold higher concentrations of different ions.

The influence of other ions, such as Na^+^, K^+^, Fe^2+^, Mn^2+^, Zn^2+^, Mg^2+^, Ca^2+^, Pb^2+^ and Cd^2+^, was also investigated in a synthetic solution containing 100-fold higher concentrations of the above nontarget ions than of As to estimate the possible influence of interference on the stripping response of trace As(III) under optimal conditions, as shown in Figure 10. The results demonstrated that the addition of nontarget ions had no significant effect on the As(III) stripping responses of the proposed electrode because the current mean values obtained in the presence of nontarget ions were within the ±2 × standard deviation range of the current measured in the absence of nontarget ions.

### 3.8. Application to Real Sample Analysis

To investigate the practical application of the proposed sensor for the determination of As(III), several soil samples were analyzed under optimal conditions. Hydride generation atomic fluorescence spectrometry (HG-AFS) was used to verify the accuracy of the prepared electrochemical platform. The pretreatment process for the soil samples is described in Section 2.4, and the analysis was carried out based on a standard addition method. The average recovery of As(III) was 98.97%, which demonstrated that the detection results obtained using the proposed platform were consistent with those obtained by HG-AFS (Table 2). To further verify the significant difference in accuracy between the proposed platform and HG-AFS, a paired t-test at the 95% confidence level was performed, and the results are presented in Table 2. Based on the statistical analysis results, we easily concluded that there was no significant difference between the two techniques because the t_calculated_ of As(III) was below t_critical_ (4.30 at 2 degrees of freedom). Therefore, the proposed modified electrode can be used for the analysis of As(III) at trace levels in real samples.

## 4. Conclusions

In this study, rGO-Au_nano_/GCE was created by a one-step electrodeposition process with CV and used for the sensitive detection of As(III). The rGO-Au_nano_/GCE sensor exhibits a better limit of detection than other Au_nano_-based modified electrodes and is more controllable and stable. Additionally, the morphology, physical and chemical properties of the fabricated rGO-Au_nano_/GCE were characterized by SEM, XRD, SWASV, EIS and CV. Parameters such as the deposition time, number of sweep cycles and deposition potential were optimized. The proposed rGO-Au_nano_/GCE sensor shows very high sensitivity for the determination of As(III) based on the combined effects of rGO and Au_nano._ The rGO produced during electrodeposition effectively increases the specific surface area and electron transfer capability of the electrode. Additionally, rGO prevents the agglomeration of Au_nano_, allowing good dispersal of Au_nano_ on the electrode surface and further improving the catalytic performance. A satisfactory recovery of As(III) in a soil sample analysis was obtained, which demonstrated that the rGO-Au_nano_/GCE sensor may have potential promising applications for As(III) monitoring of environmental and food samples.

## Figures and Tables

**Table 1:** Comparison of different modified electrodes for the determination of As(III).

Electrodes	Technique	Linear Range (μg/L)	Detection Limit (μg/L)	Reference
Au-RGO/GCE	ASLSV	0.3–20	0.1	[14]
AuNP/BDD-modified electrode	SWASV	100–1500	20	[33]
nanoPt-Fe(III)/MWCNT/GCE	ASV	0–225	0.75	[34]
rGO-Fe_3_O_4_/SPCE	SWASV	2–20	0.3	[35]
Gold nanoparticle/GCE	ASV	0–15	0.25	[36]
AuNP-PCWEs	SWASV	2–50	2.2	[37]
rGO/Fe_3_O_4_/GCE	SWASV	0.1–20	0.12	[38]
Gold disk	SWASV	225–1800	3.7	[39]
Sub-BT/Au	DPASV	0–11.25	0.28	[40]
Nano-Au/GCE	LSV	3.675–87.075	1.8	[41]
I^−^-nano-Au/PANI/GCE	SWV	610–3050	0.4	[42]
rGO-Au_nano_/GCE	SWASV	1–60	0.08	This work

ASLSV: anodic stripping linear sweep voltammetry, AuNP/BDD: gold nanoparticles on boron-doped diamond, MWCNT: multiwalled carbon nanotube, AuNP-PCWEs: gold nanoparticle-modified paper-based carbon working electrodes, rGO: reduced graphene oxide, BT: butanethiol, PANI: polyaniline.

**Table 2:** Results of the detection of As(III) in soil sample extracts.

Sample No.	Added (μg/L)	Detected by SWASV ^a^ (μg/L)	Detected by HG-AFS ^a^ (μg/L)	*t*_calculated_	Recovery (%)
1	-	13.57 ± 0.58 ^b^	13.69 ± 0.22 ^b^	1.95	-
	5.00	18.63 ± 0.74			101.20
	10.00	23.42 ± 0.60			98.50
2	-	18.62 ± 0.52	18.73 ± 0.23	1.73	-
	10.00	28.43 ± 0.49			98.10
	15.00	33.49 ± 0.62			99.13
3	-	15.38 ± 0.63	15.44 ± 0.41	1.62	-
	15.00	30.13 ± 0.41			98.33
	20.00	35.09 ± 0.73			98.55

^a^ SWASV and HG-AFS measurements were repeated five times (n = 5). ^b^ Mean value ± standard deviation.
